# Dendrimers as Non-Viral Vectors in Gene-Directed Enzyme Prodrug Therapy

**DOI:** 10.3390/molecules26195976

**Published:** 2021-10-01

**Authors:** Adriana Aurelia Chis, Carmen Maximiliana Dobrea, Luca-Liviu Rus, Adina Frum, Claudiu Morgovan, Anca Butuca, Maria Totan, Anca Maria Juncan, Felicia Gabriela Gligor, Anca Maria Arseniu

**Affiliations:** Preclinical Department, Faculty of Medicine, “Lucian Blaga” University of Sibiu, 550169 Sibiu, Romania; adriana.chis@ulbsibiu.ro (A.A.C.); carmen.dobrea@ulbsibiu.ro (C.M.D.); liviu.rus@ulbsibiu.ro (L.-L.R.); anca.butuca@ulbsibiu.ro (A.B.); maria.totan@ulbsibiu.ro (M.T.); ancamaria.juncan@ulbsibiu.ro (A.M.J.); felicia.gligor@ulbsibiu.ro (F.G.G.); anca.arseniu@ulbsibiu.ro (A.M.A.)

**Keywords:** GDEPT, GDEP therapy, non-viral vector, dendrimer, delivery vehicles, gene delivery system, transgene, targeted therapy

## Abstract

Gene-directed enzyme prodrug therapy (GDEPT) has been intensively studied as a promising new strategy of prodrug delivery, with its main advantages being represented by an enhanced efficacy and a reduced off-target toxicity of the active drug. In recent years, numerous therapeutic systems based on GDEPT strategy have entered clinical trials. In order to deliver the desired gene at a specific site of action, this therapeutic approach uses vectors divided in two major categories, viral vectors and non-viral vectors, with the latter being represented by chemical delivery agents. There is considerable interest in the development of non-viral vectors due to their decreased immunogenicity, higher specificity, ease of synthesis and greater flexibility for subsequent modulations. Dendrimers used as delivery vehicles offer many advantages, such as: nanoscale size, precise molecular weight, increased solubility, high load capacity, high bioavailability and low immunogenicity. The aim of the present work was to provide a comprehensive overview of the recent advances regarding the use of dendrimers as non-viral carriers in the GDEPT therapy.

## 1. Introduction

During the research process for the development of new therapies, different strategies have been addressed, including prodrugs that can be transported and activated in the vicinity of targeted areas, combined with certain genes, enzymes or proteins, for an accurate and effective delivery of the drug to the target site or at the level of tissues or receptors, thereby increasing the selectivity of drugs for specific cellular targets, and consecutively increasing their therapeutic effect and decreasing the incidence of side effects.

Selective activation of a drug at the level of the target tissue by exogenous enzymes or enzymes expressed at the target site can be achieved through the following strategies, using prodrugs and various vectors [1,2,3].

- Antibody Directed Enzyme Prodrug Therapy (ADEPT) = the enzyme is vectorized by antibodies or catalyzed directly by antibodies, towards antigens expressed on tumor cells [4,5].
- Virus Directed Enzyme Prodrug Therapy (VDEPT) = uses viral vectors to provide the gene encoding an enzyme which can trigger the activation reaction of a cytotoxic agent following systemic administration [6,7].
- Gene Directed Enzyme Prodrug Therapy (GDEPT) = uses a gene encoding an enzyme that is delivered at the target site through a carrier. The terminology of Genetic Prodrug Activation Therapy (GPAT) [1] is also used in the literature.
- Polymer Directed Enzyme Prodrug Therapy (PDEPT) = passive targeting approach using a prodrug-conjugated polymer, followed by the administration of an enzyme-polymer conjugate, to achieve site-specific activation [8].

**Figure 1:**
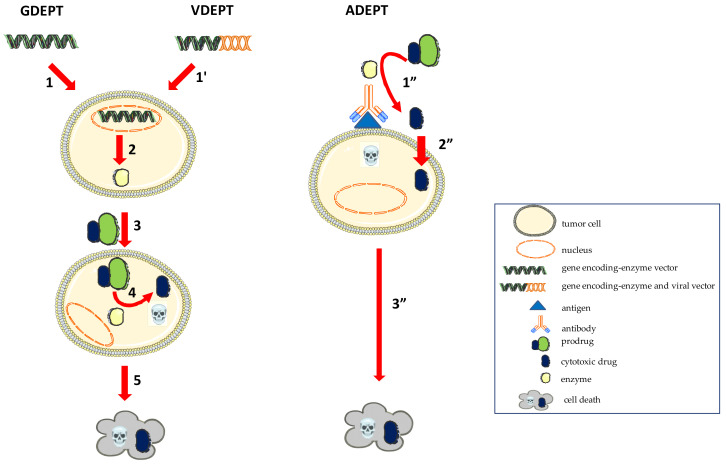
Diagram of ADEPT, VDEPT and GDEPT therapeutic strategies. 1−The gene encoding an enzyme that is delivered at the target site; 2−The triggering of the intracellular expression of the enzyme; 3−Systemic administration of a prodrug; 4−The activation of an inactive prodrug into an active cytotoxic agent; 5−The death of the transduced tumor cells; 1’−Viral vectors providing the gene encoding an enzyme; 1”−The enzyme that is vectorized or catalyzed directly by antibodies, towards antigens expressed on tumor cells, activating the prodrug; 2”−The cytotoxic effect of the active drug that entered into the cells; 3”− The death of tumor cells.

Different approaches for the targeting and release of an active drug molecule are schematically presented in Figure 1 [9].

The current review addresses the GDEPT strategy with the presentation of vectors used for the targeting and release of active molecules. Furthermore, this research provides an overview of the involvement of dendrimers in specific vectorization for GDEPT therapy, with an emphasis on their main advantages. The cytotoxicity of dendrimers is presented, together with different structural modulation strategies that have been studied in the scientific literature to improve their safety profile and efficacy. 

## 2. GDEPT Strategy

Gene therapy is an important advance in improving the specificity of a drug. The main goal of this strategy is to provide a gene encoding the drug activation enzyme or a functional protein inside the targeted tissues, followed by systemic administration of the substrate drug [10,11]. 

With the onset of this kind of therapy, new approaches have been made for therapeutic purposes and they are much more effective than conventional strategies for treating cancers, genetic diseases, including cystic fibrosis, and other diseases, such as cardiovascular and nervous diseases and complex autoimmune diseases. In addition, the gene therapy approach can also be used for the treatment of acquired diseases or polygenic ones, by increasing the immune response using complex gene delivery strategies, releasing suicide genes that trigger cell apoptosis [12,13,14,15]. Additionally, gene therapy is increasingly being addressed in the fundamental research and in bio-nanotechnology to investigate the basic cellular mechanisms or obtain specific proteins [16,17,18].

GDEPT uses transgenes encoding enzymes that can transform a non-toxic prodrug into a cytotoxic agent. This is a promising strategy to limit systemic toxicity and improve the selectivity of chemotherapy.

The GDEPT concept was introduced over two decades ago, but its therapeutical importance in clinical practice has emerged in the last decade, with an increasing number of GDEPT systems entering clinical trials [19,20]. With the rapid progress in molecular biology and biotechnology, chromosomal defects have been identified for a large number of human hereditary diseases and the prospects for healing using gene therapy have been considerably increased. 

**Figure 2:**
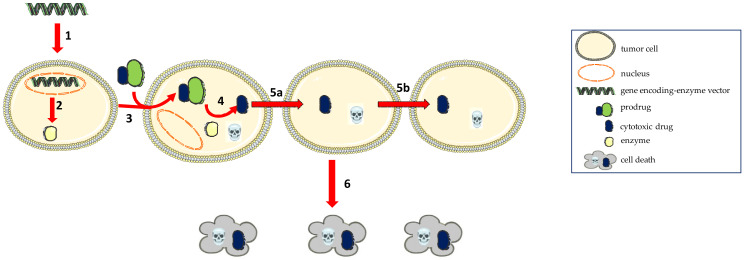
General representation of the GDEPT strategy involving the occurrence of the bystander effect. 1−The gene encoding an enzyme, that is delivered at the target site; 2−The triggering of the intracellular expression of the enzyme; 3−Systemic administration of a prodrug; 4− The activation of an inactive prodrug into an active cytotoxic agent, that leads to the transduced tumor cell death; 5a, 5b−The transfer of active metabolites to non-transduced neighboring cells; 6−The destruction of distant cells.

Gene therapy was first introduced by Joshua Lederberg in 1963 [21], but research in human genetics was not really addressed until the 1980s. Subsequently, the first clinical study on gene transfer was conducted by Anderson et al. in 1990 [22] for severe combined immunodeficiency (SCID). GDEPT as a therapeutic strategy is designed as a three-component system: an inactive drug (prodrug), a gene encoding an enzyme that turns the inactive precursor into an active drug and a carrier, also known as a vector (Figure 2).

The presence of the bystander effect contributes to the therapeutic effect of the GDEPT strategy [23], causing the destruction of neighboring cells of the transduced cell. Moolten (1986) has elucidated this phenomenon [24] and suggested that transduction, even in only 10% of the cells, is sufficient to destroy the entire population of tumor cells [25,26,27]. This effect is mediated by the transfer of toxic metabolites from transduced cells to non-transduced cells, through a passive or active mechanism. The bystander effect causes the death of the neighboring tumor cells by amplifying or extending the cytotoxic activity of the drug. Two categories of action are important in the emergence of the bystander effect: a locally mediated action and an immune response [18].

Regarding the local effect, apoptosis of the neighboring cells is due to the transfer of the cytotoxic drug through gap junctions, apoptotic vesicles or the diffusion of soluble toxic metabolites [27,28]. The lipophilic character of the activated drug is an important parameter in the occurrence of the bystander effect generated by passive diffusion [29]. Diffusion is quantitatively described by Fick’s laws and the Stokes–Einstein equation and its magnitude is directly proportional to the diffusion coefficient (D). Usually, in cancer treatment, large molecules (with small D values) are involved and low diffusion rates are achieved. Since cytoplasm is a complex gel (with structural obstacles) and a large-value media viscosity, a small value of D and an altered diffusion rate are expected. Furthermore, passive diffusion is influenced by partition coefficient (Pc), acidity constant (Ka) and pH differences between donor and receiver zones. Molecules with log Pc > 3.5 tend to remain in lipid bilayers and will not reach cytosol and molecules with log Pc < 0 will remain in extracellular fluids and are not able to pass through cell membrane. Considering acidic and basic molecules, only unionized forms can passively diffuse through cell membranes. In tumors, extracellular pH is acidic while intracellular pH is neutral to alkaline, so the uptake of basic substances is diminished [30,31].

The delivery and distribution of genes or nucleic acids (NAs) to target cells, a process called transfection, is a new breakthrough in molecular medicine for the treatment of many diseases [15,32].

In gene therapy, to achieve the desired therapeutic efficacy, the genetic material must be directed and transported to the selected tissue at an increased level of specificity.

Gene delivery, through this therapeutic approach, consists of modulating gene expression patterns by delivering exogenous genetic material at the target site, in the following variants:

(i) Chimeric circular plasmid DNAs (pDNA - plasmid DNA is a small molecule of physically separated DNA and distinct from chromosomal DNA, commonly used for gene duplication, transfer, and manipulation), which are hybrid plasmids containing a specific gene of interest (e.g., a gene encoding an easy-to-follow protein, such as luciferase or a fluorescent protein) [33];
(ii) Messenger RNA (mRNA);
(iii) Short RNA fragments, such as: siRNA, short-interfering RNA or rate-reducing RNA, is a double-stranded RNA that provides interference between gene expression and complementary nucleotide sequences; miRNA, a non-coding RNA molecule that functions by regulating the expression of post-transcription genes; short hairpin RNAs;
(iv) Antisense oligonucleotides (ASO) at the targeted site of action [34,35,36].

This therapeutic strategy is characterized by several major advantages:

(i) Specificity and selectivity: gene expression can be controlled by tumor cell-specific promoters/specific antigens, enzymes and their associated reactions, and can be directed into tumor cells without affecting healthy cells [37,38]. Due to the preferential conversion of the prodrug into the active drug in transfected tumor cells, with a minimal impact on healthy cells, the therapeutic index of the precursor is generally much higher than in conventional antineoplastic chemotherapy [39]. A wide range of tissue-specific promoters have been developed, such as: the human telomerase reverse transcriptase (hTERT) promoter, the carcinoembryonic antigen (CEA) promoter, the osteocalcin (OC) promoter [40]. More recently, other gene promoters have been developed, such as: auxin response factors (ARF), glucose-regulated protein (GRP78), CXC chemokine receptor-4 (CXCR4), osteopontin (OPN) [40,41,42,43,44]. Efficacy has been demonstrated for all of these promoters, but the only one to enter clinical trials was hTERT [45]. The main challenge is the low transcription profile of promoters, which causes the expression of suicide gene at a low level, which is insufficient to convert an optimal fraction of prodrug into cytotoxic molecules. To overcome these obstacles, chimeric and artificial promoters are increasingly studied [46,47].
(ii) Another advantage of this therapeutic strategy compared to conventional therapy is the presence of the bystander effect, which is especially applied in antineoplastic therapy [24].

### 2.1. Gene Distribution and Delivery Vectors in GDEPT

The main purpose of gene therapy is represented by the delivery and use of genes, especially nucleic acids, which must act intracellularly, but most nucleic acids are rapidly degraded after systemic administration and do not easily cross the plasma membrane. Transport vectors contribute, sometimes decisively, to the effectiveness of this therapeutic strategy [48]. Numerous strategies and tools have been used for the intracellular delivery of nucleic acids, including viral (VV) and non-viral vectors (nVV), with the latter being represented by chemical vectors (cationic lipids, lipid polymers, cationic polymers) and physical vectors obtained by different methods (DNA bombardment, ultrasound, electroporation, sonoporation, magnetofection) [49,50,51].

The use of revolutionary techniques focusing on clustered regularly interspaced short palindromic repeats (CRISPR) is becoming more and more evident. The CRISPR/Cas9 system has already been customized to provide new defense capabilities to the host organism, either by modifying the host genome or by directly targeting the factors used [50,52]. The CRISPR/Cas9 system offers great therapeutic potential. The method of delivery through this technology determines the efficiency of genome modification, but also the frequency of unwanted effects or the occurrence of other effects, effects outside the objectives. The delivery vectors used by this technology are VVs and nVVs [53,54]. 

VVs, which can transfer genetic material to the host cell, are biological systems derived from natural viruses that have been modified to increase safety, specific absorption, and to improve the efficiency of the active drug molecule. The VVs used in GDEPT are as follows: retro and adenoviruses, herpes simplex virus (HSV), or adeno-associated virus (AAV) and poxvirus [55,56]. The most commonly used DNA VVs are based on adenoviruses and AAV. These vectors can be transformed into efficient gene delivery systems by replacing part of their genome with a therapeutic gene.

From the beginning of the development of gene therapy, VVs have evoked interest due to their high efficacy in the administration of genes both in vitro and in vivo [57,58]. Subsequently, AAV has been widely studied and become one of the most selected VVs for the development of gene therapy due to its structure and safety profile. Additionally, recombinant AAV (which lacks viral DNA) has proved its efficacy and safety throughout different stages of testing, including clinical trials [59]. Furthermore, several therapeutic strategies based upon lentiviral vector-engineered cells are in late-phase development and target hematopoietic stem cells [60]. The major limitation of VVs is their capacity to transport a relatively small amount of genetic material, no greater than 5kb [61]. Retroviruses, for example, are associated with oncogenic risks related to insertional mutagenesis, leading to the development of malignant cells [62]. Moreover, repeated administration of viral vectors can cause a number of inflammatory or immune responses [63].

nVVs, on the other hand, have received increasing attention and remarkable interest due to their improved safety, greater flexibility, and ease of synthesis. These nVV systems have more advantages over VVs, such as decreased immunogenicity and toxicity, higher specificity for the target cell, can be more easily modulated, and can have higher productivity [64]. On the other hand, the lower transfection efficiency of a nVV is restricting the use of this vector system compared to a VV [65]. The primary factor related to the transfection efficiency of nVV is represented by the anatomical barriers. A decreased cell adhesion and hemolysis can also contribute to low transfection [66]. The intracellular uptake by the target cell is considered a key factor in transfection efficiency [67]. In addition, the lower transfection efficiency may be related to the lack of dissociation of the polyplexes and the inability to enter the nucleus [68].

**Figure 3:**
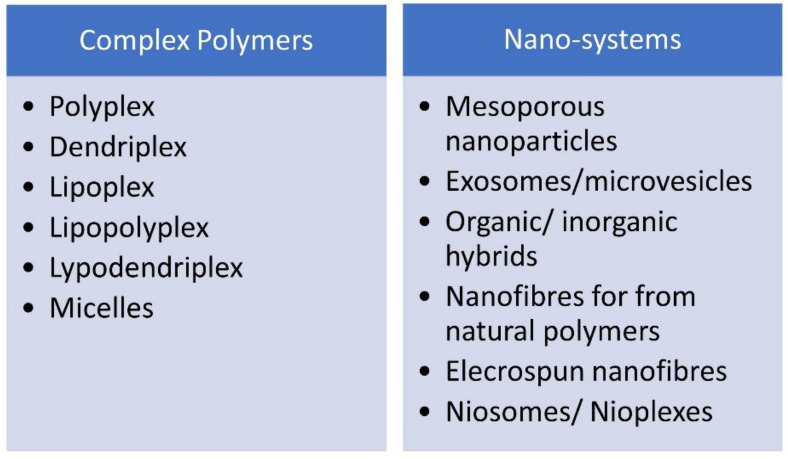
Examples of nVV systems for gene delivery in GDEPT.

There are several types of nVVs, commonly used in GDEPT, such as: polyplexes - short-lived electrostatic complexes that require an excess of polymer [69]; dendriplexes [70], lipoplexes [71], lipopolyplexes [72], lypodendriplexes [73], and micelles - dynamic amphiphilic polymers [74]. Nano-systems include: mesoporous nanoparticles [75,76], exosomes / microvesicles [77], organic / inorganic hybrids [78,79], nanofibres for from natural polymers [80,81], elecrospun nanofibers [82], niosomes / nioplexes [83], etc. (Figure 3).

Niosomes represent a special category of nVV used in gene therapy, characterized by their stability and low toxicity and relatively easy to obtain. Niosomes are characterized by the existence of three main components: a nonionic surfactant that improves the long-term stability, a cationic lipid component responsible for electrostatic interactions with negatively charged genetic material, and a different component, which is useful for improving physicochemical properties and biological efficiency [83,84]. 

Polymersomes studies offer new opportunities for advancement in gene therapy and in the delivery of various protein molecules [85]. Polymersomes are stable polymeric vesicles, consisting of amphiphilic polymers of different molecular weights, which improve the efficiency of drug administration. They are more stable compared to liposomes and have a lower immunogenicity in vivo. Their ability to encapsulate both hydrophilic and hydrophobic drugs, biocompatibility, robustness, high colloidal stability and simple methods for conjugating ligands make these polymers promising candidates for the administration of therapeutic drugs in cancer therapy [86].

Polyplexes are commonly used for nucleic acid release, especially polyplexes formulated with cationic polymers. These complexes are formed spontaneously by entropic electrostatic interactions between positively charged polymers, containing ionizable amino groups (N) and negatively charged nucleic acids containing phosphate groups (P). For gene delivery, an excess of polymer (with an N/P ratio > 1) is usually used to increase the load of nucleic acid, generating delivery vehicles with a positive surface charge [48,87].

nVV systems made up of complexes of cationic lipids, cationic polymers (polyplexes) can protect DNA from the degradation caused by specific enzymes (nucleases) and deliver them directly to the target cells [88]. However, cationic liposome complexes, also known as lipoplexes [89], have some limitations in gene delivery, especially in terms of difficulty obtaining reproducible liposomes and lipoplexes, in vitro and in vivo toxicity, and colloidal instability after systemic administration [90].

Most studies on cationic polymers focus on addressing the problems associated with low transfection efficiency, cytotoxicity and nonspecific cell absorption [91].

A major challenge in the GDEPT strategy is represented by the endosomal barrier that influences transfection, as polymers used as vectors often remain trapped inside endosomes, where some of them can be degraded and only some of the particles are able to achieve endosomal escape [92,93].

Some theories supporting the effectiveness of nVV in the GDEPT strategy have been put forward to explain the endosomal escape, such as [94]:

(i) “Proton sponge theory”: first issued around the 1990s [95,96]. Following the protonation of polymers, their chain elongates due to electrostatic repulsion. Thus, it has been shown that the expansion of the space occupied by the polymer can contribute to the increase in the so-called “umbrella hypothesis” [97,98];
(ii) Membrane destabilization: it has been shown through molecular dynamics that, by elongating the chains of some polymers (such as polyethylenimine), they might be able to interact with the endosomal membrane, leading to the formation of hydrophilic pores in the membrane lipid bilayer. Thus, the lipid bilayer can destabilize, contributing to the release of the polymer from the endosomal space [99,100];
(iii) Increasing the volume of polymers, as an additional factor that could influence the endosomal escape of polyplexes [101,102].

The proton sponge effect is currently considered to be preceded by an initial membrane destabilization induced by the positive charge of the polymer, followed by membrane destabilization as a consequence of the “umbrella hypothesis” [103].

Table 1 shows some selective and representative examples of polymers used in GDEPT as nVVs.

### 2.2. Gene Delivery Mechanisms through nVV

The gene delivery through which the exogenous NA enters the cell takes place in the nucleus for pDNA (plasmid deoxyribonucleic acids) and in the cytoplasm for siRNA (small interfering ribonucleic acids) or miRNA (microRNAs). The exact mechanism of gene delivery through nVV is not fully elucidated [124,125]. The following stages are generally presumed, in which several biological barriers are overcome, namely: (i) cell membrane that determines cell absorption, (ii) inclusion in the endosomal system, (iii) nuclear translocation through the nuclear envelope and (iv) cellular transcription/translation [126,127].

**Figure 4:**
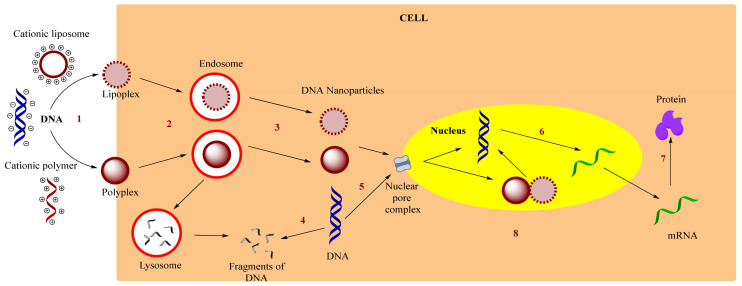
The basic mechanism of nVV gene release through polyplex and lipoplex. (1) condensation; (2) endocytosis; (3) endosome escape; (4) degradation of DNA; (5) cytosolic migration; (6) transcription; (7) translation; (8) nuclear translocation. Adapted from [66], published J Transl Med, 2018.

Vectorization of DNA occurs through various interactions involving cationic entities: a polymer or a liposome that enables the endocytosis. After evacuation of the endosomes and proper transcription and translation, the specific proteins for biological effects are produced. Figure 4 schematically illustrates this mechanism [124].

Immediately after establishing direct contact between non-viral vector/gene complex (nVGC) and target cells, interactions are triggered on the cell surface. Cellular absorption of nVGC takes place through endocytic or non-endocytic pathways, endocytic pathways, such as phagocytosis and endocytosis, which is more frequently addressed in non-viral gene delivery. One of the most studied cellular uptake mechanisms of nVV is represented by clathrin-mediated endocytosis. Alongside this, clathrin-independent endocytosis includes caveolae-mediated endocytosis, flotillin-dependent endocytosis, the adenosine diphosphate-ribosylation factor 6 (ARF6)-dependent endocytosis, the RhoA-dependent pathway and the GTPase regulator associated with focal adhesion kinase-1 (GRAF1)-dependent endocytosis [128]. Macropinocytosis occurs through the formation of actin-driven extensions of the plasma membrane that form cup-shaped ruffles [129]. On the other hand, the non-endocytic pathways include microinjection, permeabilization using pore-forming reagents and electroporation [130]. 

The intracellular trafficking includes the endosomal escape, vector unpacking and, only for DNA, the nuclear entry [61]. Thus, through the cellular absorption mechanism nVGC becomes partially encapsulated and dissociates to form intracellular vesicles, and finally integrates into endolysosomes. The nVGC formations encapsulated in the form of intracellular vesicles in the first stage enter the cell, from which they are released into the cytoplasm through a process called endosomal escape. Specifically, pDNA can cause an increase in osmotic pressure in the endosomes, leading to disruption of the endosomal membrane, and thus releasing nVGC into the cytoplasm.

Nuclear translocation, in the form of nVGC or pDNA, can be achieved by cell-dependent or cell-independent pathways. In the cell-dividing-dependent pathway, nVGC, such as polyplexes enter the nucleus when the nuclear envelope decomposes during the growth and preparation for mithosis/mithosis (G2/M) transition stage of cell division. Transcription is the process of RNA biosynthesis that takes place in the nucleus based on the DNA template, initiated by the interaction of the transcription factor in the nucleus and the delivered pDNA promoter. The next step is to transport the mRNA out of the nucleus to the cytosol to be translated into the corresponding protein [65].

## 3. Dendrimers in GDEPT Strategy

### 3.1. History of Dendrimers

**Figure 5:**
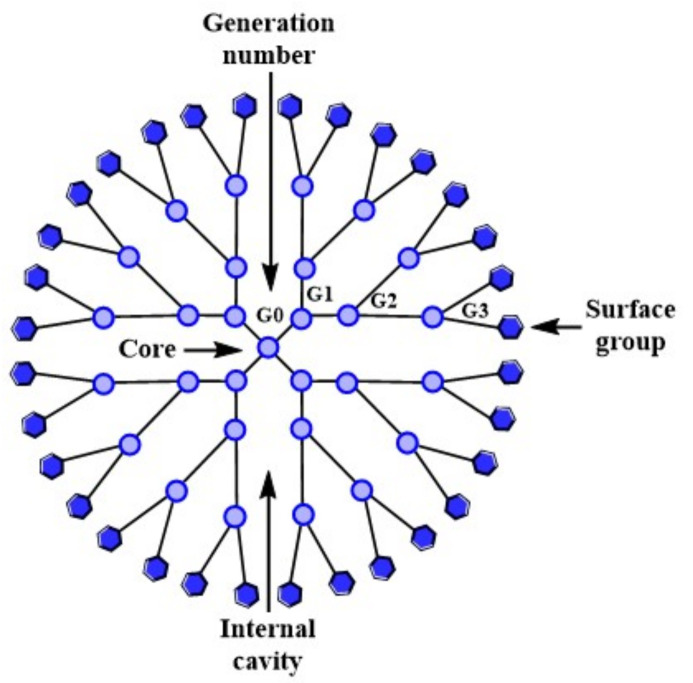
General structure of dendrimers. Adapted from [139], published by Int. J. Nanomed, 2009.

Dendrimers (Greek: dendron = tree; meros = parts, branches) [131], arborols or cascade molecules, are an architectural three-dimensional class of globular polymers [132,133,134]. Their structure is well-defined, homogenous and mono-dispersed, and consists of three parts: 1) central part (core/nucleus); 2) amplification region formed by branches or arms and 3) peripheral part with surface functional groups (Figure 5) [132,133,134,135,136,137,138].

**Figure 6:**
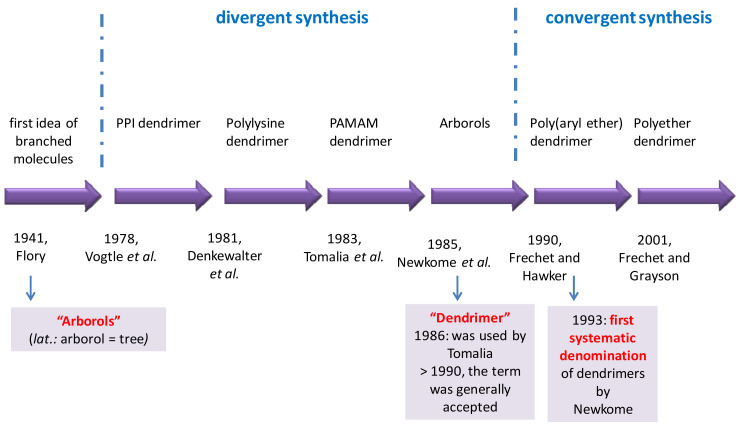
Schematic history of dendrimers [132,133,134,140,141,142,143].

The first idea regarding the synthesis of dendritic structures was elaborated by Flory in 1941, and the first synthesis of this type of polymers was realized in 1978 by Vogtle et al. [140]. They used a divergent strategy to create dendrimers through the following steps: (i) creating the nucleus (core); (ii) coupling of the monomer; (iii) creating the reactive surface for a new monomer’s coupling; (iv) amplification of the latter two steps depending on the number of generations desired [132]. After 1990, a new approach to dendrimer synthesis (convergent synthesis) was registered. Frechet and Hawker first created the peripheral surface of the dendrimers, and then continued towards the nucleus, creating dendritic segments by coupling the monomers (Figure 6) [132]. 

The dendritic design allows for the presentation of the peptides in a three-dimensional, branched way, resulting in a globular shape [144], and thus imitating a globular protein. Unlike other classes of dendrimers, the peptide ones are used in biomedicine due to their biological origin [145].

### 3.2. Advantages of Dendrimers and Nanoparticles in the Distribution and Release of Drug Molecules at the Site of Action

Dendrimers or dendritic polymers can act as drug-delivery systems [140]. The use of nanotechnology in medicine and the administration of drugs is constantly growing. Pharmaceutical companies use NPs to reduce toxicity as well as reduce the side effects of drugs [118,146]. In addition to reducing toxicity and side effects, an important role is played by the targeting and delivery of medicines, leading to increased efficacy [118]. NPs are attractive for medical purposes because of their unique characteristics, namely: the ratio of surface area to mass is much higher than in the case of other particles. NPs also have a relatively large functional surface, which is able to bind, absorb and transport other compounds, such as proteins and drugs. NPs have dimensions below 100 nm, but in certain targeted delivery areas, relatively large NPs (> 100 nm) are required to load a sufficient amount of drug on the particles. The modified particles are used to deliver the drug, but the drug itself can also be formulated on a nano-scale, functioning as its own carrier [146]. Thus, in pharmaceutical technology, these polymers are used as excipients in the development of various pharmaceutical forms, for transporting cancer drugs, cardiovascular drugs, antibiotics, anti-inflammatory drugs, antivirals, anti-glaucoma agents, contrast agents, etc. [118,132,140]. 

NPs possess the ability to cross the blood–brain barrier, opening a passage for the distribution of drugs in the brain. Their nanosize allows access to different cell compartments, even in the nucleus. Dendrimers offer advantages such as: nanoscale size, increased solubility, high load capacity, high bioavailability, increased absorption and efficiency, precise molecular weight, internal cavities available for loading and low toxicity [118,147] (Table 2). These attributes give dendrimers the ideal property of being an active excipient by increasing the solubility of poorly water-soluble drugs [118,148]. Dendrimers are also excellent vehicles for the efficient and safe transport of drugs [118]. They have a low viscosity, with this property being advantageous for the preparation of drugs because it facilitates the immediate release of drugs [118].

One advantage of polymer-conjugated drugs is the fact that they have a prolonged half-life, greater stability, are soluble in water and have low antigenicity and immunogenicity [134]. Dendrimers also have less advantageous properties, which prevent their large-scale use. 

The limited drug incorporation into dendrimer cavities, cytotoxicity and inability to control the rate of drug release are among their disadvantages [118]. For example, in vivo, the use of PAMAM-NH_2_ was negatively influenced by their strong cytotoxicity [154].

### 3.3. The Efficiency of Dendrimers in GDEPT 

In the GDEPT therapeutic strategy, the use of dendrimers as delivery vectors has proved to be a promising and effective strategy in cancer therapy, due to obvious advantages, such as: three-dimensional structural conformation, hydrosolubility, low immunogenicity and high functionalization [155]. The specific structural features with numerous ramifications [156,157] have determined the wide use of dendrimers for the delivery of active drug molecules [158], but also for the development of nanoplatforms for various antineoplastic therapies.

The use of dendrimers in GDEPT is favorable for the targeted delivery of genes because NAs can generally easily form NP cationic complexes with different dendrimers, through an electrostatic compression mechanism, due to the presence of numerous amine groups. [159].

Through the dendritic ramifications, these complexes ensure the protection of NA from enzymatic degradation and, moreover, different dendrimers containing numerous tertiary amine groups show the “proton sponge” effect, which protects complexes from the action of lysosomes, endosomal and nuclear gene degradation [13,160].

The efficiency of gene transfection depends on several factors: dendrimer type and cellular parameters on both sides of the membrane. For the optimal delivery of the gene, some characteristics of the dendrimers are important, such as: size, conformation, functionality and ramification of the surface, the number of generations, and the physico-chemical characteristics [161].

In vivo, there are many barriers that prevent the genes entering the cell. Therefore, when designing dendrimer delivery systems, it is necessary to account for both the mechanisms of gene delivery through dendriplexes and the use of effective strategies to overcome physiological barriers by designing an appropriate type of dendrimer with surface or base changes [89].

Successful in vivo gene delivery studies via dendriplexes were reported. The brain is the organ which elicits the most interest, and possesses many challenges due to the blood–brain barrier. Some of the research has showed encouraging results regarding luciferase gene expression [162,163,164], β-galactosidase [162,163], downregulation of the epidermal growth factor receptor gene [165], expression of plasmids targeting CCL20 (a chemokine mediating neuroinflammation) and its sole receptor CCR6 [166]. Other targeted organs or systems were as follows:

(i) Lungs: regulation of chloramphenicol-l-acetyltransferase expression [167], regulation of human factor VIII gene expression [168], expression of green fluorescent protein [73,169,170];
(ii) Liver: anti-sense oligodeoxynucleotides [171], regulation of human factor VIII gene expression [168], expression of green fluorescent protein [73,169,170], - suicide gene therapy of hepatocarcinoma [172];
(iii) Spleen: regulation of human factor VIII gene expression [168], expression of green fluorescent protein [73,169,170], luciferase [172];
(iv) Kidney: regulation of human factor VIII gene expression [168], expression of green fluorescent protein [73,169,170];
(v) Heart: expression of green fluorescent protein [73,169,170];
(vi) Prostate: plasmids encoding TNFα [173,174];
(vii) Ovary: siRNA that targets p70S6K in cancer stem cells [175];
(viii) Skin: plasmid DNA encoding p73- inhibition of tumor growth for animals with A431 and B16 tumors [176];
(ix) Blood: positive effects in acute myeloid leukemia using pivotal tumor-suppressor and negative regulator of FLT3 gene for the expression of tyrosine kinases 3 receptor [176].

**Figure 7:**
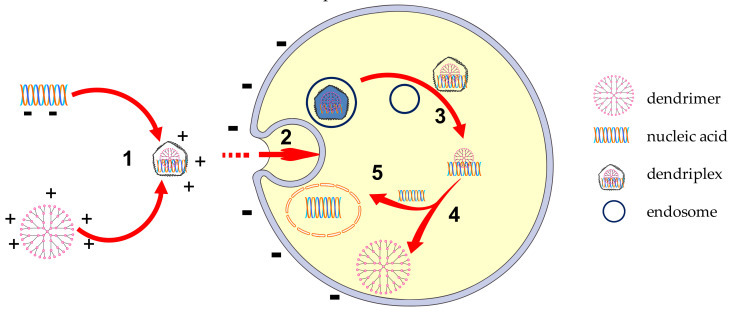
Mechanism of gene transfection through cationic dendriplexes. (1) nucleic acid condensation with dendrimers forming stable dendriplexes; (2) cellular uptake of dendriplex; (3) endosome escape of dendriplex; (4) the release of the dendriplex components; (5) the entrance of the nucleic acid in the nucleus. Adapted from [13], published by Elsevier, 2020.

Figure 7 schematically represents the mechanism of the therapeutic gene release from the cationic dendriplex.

**Figure 8:**
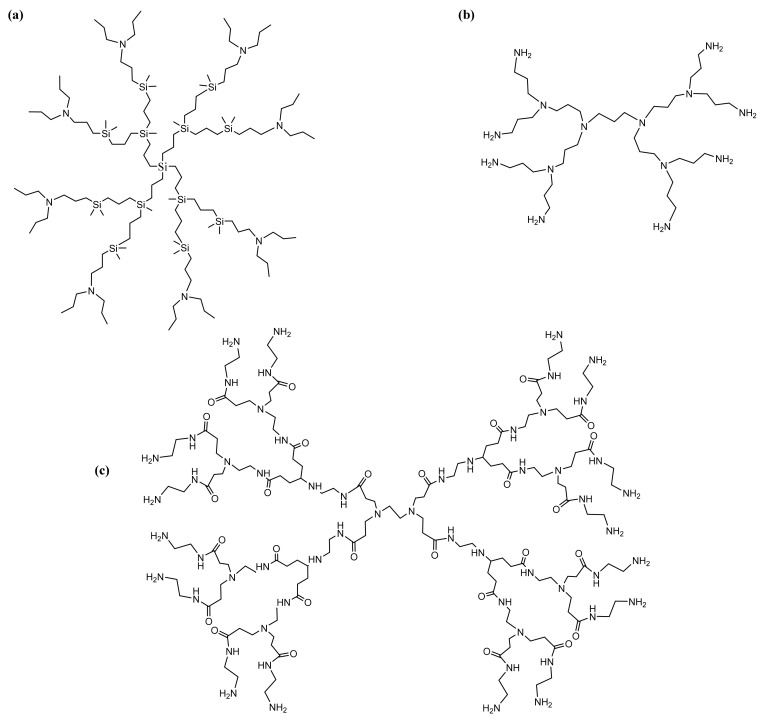
General structures of some G2 dendrimers: (**a**) carbosilane dendrimers; (**b**) polypropylene-imine dendrimers; (**c**) cationic poly(amidoamine) dendrimers, most commonly used to provide therapeutic nucleic acids. Adapted from [184], published by Pharmaceutics, 2018.

PAMAM dendrimers [177,178,179], phosphoric dendrimers [180,181], PPI dendrimers [182,183] are among the most-used dendrimers in GDEPT. Figure 8 presents the most-used dendrimers for the delivery of therapeutic genes [182].

### 3.4. In Vitro and In Vivo Studies on the Efficacy of Dendriplexes

Several studies focused on the gene therapy using dendrimers as the main carriers of genetic material. There are some experimental studies which report the efficacy of dendrimers in cancer therapy. Recent publications have presented clinical trials on the use of dendrimers in therapy [70,185]. Accounting for the results of in vitro and in vivo studies, clinical trials regarding the use of dendrimers as nVV in GDEPT strategy represent the next phase before therapeutical use.

In a few in vitro studies on human brain cell lines, dendrimers with anticancer molecules such as temozolomide, 5-fluorouracil (5-FU), and doxorubicin were used. Thus, in a study on human brain glioma cell line U251, a co-delivery system of PAMAM with antisense-miR-21 oligonucleotide and 5-FU improved the cytotoxicity of the drug, increased the apoptosis of U251 cells, and decreased the migration ability of the tumor cells [186]. Another study performed on glioma cell lines presented favorable effects (cell apoptosis and reverse drug resistance) after the usage of a microRNA-21 inhibitor and temozolomide included in a dendritic structure [187]. A study on LN18 glioblastoma cells reported that a prodrug with doxorubicin modified with a galactose unit through a self-immolative group and loaded in a dendrimer-like mesoporous silica nanoparticles, which are capped with a disulfide-containing polyethyleneglycol gatekeeper, presents a selective toxicity and a superior efficiency compared to the free prodrug [188]. 

Human antigen R (HuR, ELAVL1) is an expressed RNA-binding protein that is very highly expressed in different ovarian tumors. In a mouse ovarian tumor model, siRNAs combined with folic acid-derivatized DNA dendrimer nanocarrier (FA-3DNA) inhibited HuR expression and suppressed tumor growth and ascites development [189]. 

In another experimental model on cancer stem cells with the mark of CD44, it was observed that hyaluronic acid-modified PAMAM-entrapped gold nanoparticles delivering the METase gene suppressed the gastric tumor growth (hyaluronic acid was used to recognize the cells with the mark of CD44) [190]. 

A PAMAM derivative was used to co-deliver p53 plasmid and RG7388 (a mouse double minute 2 homolog inhibitor) to the tumor site. The study performed on MDA-MB-435 and MCF-7/WT xenograft mice model showed a synergistic action of p53 plasmid and RG7388 on cell apoptosis and inhibition of cell proliferation [191].

In a xenograft brain tumor model using U87MG cells on mice, the arginine-modified polyamidoamine (PAMAM-R) dendrimer delivered the human interferon beta gene included in a plasmid (pORF-IFN-β). The PAMAM-R/pORF-IFN-β complex induced apoptosis specifically in tumor cells [192].

Natural-killer-cell-derived exosomes express proteins with cytotoxic activity in tumor cells and non-cytotoxic activity in normal tissues. The in vivo study performed by Wang et. al showed the efficacy of nanoparticles assembled with a PAMAM dendrimer core loading gene therapeutic agents (miRNA) and a hydrophilic exosomes shell, which facilitated the tumor targeting and acted as a direct antitumor agent. The targeting and miRNA delivery were most efficient on the neuroblastoma cells [193].

Another in vivo therapy study was performed in mice bearing s.c. PC3 prostate carcinoma xenografts. It reveals a marked inhibition of tumor growth determined by extracellular vesicles-modified PEI-based nanoparticles with small RNAs (PEI/siRNA) complex [194].

Promising results were noticed in another in vivo study on a triazine-modified PAMAM dendrimer, which transfected a plasmid (the tumor necrosis factor related apoptosis inducing ligand plasmid), and subsequently inhibited the tumor cells growth in osteosarcoma induced in mice [128].

### 3.5. The Stability of Dendrimers and Dendriplexes

To determine the stability of dendrimers and dendriplexes, hydrolysis experiments in solutions with different pH values and in human plasma were conducted. The stability of dendrimers is directly influenced by the pH and their concentration [185]. Additionally, PAMAM dendrimers exhibit low stability during heat sterilization, while PPI and PLL dendrimers are thermostable [70].

A limitation related to dendriplexes stability is the interaction with several glucosaminoglycans from the extracellular matrix (heparin, heparan sulphate, chondroitin suphate and hyaluronic acid). Several dendriplexes were synthesized combining G4-PPI dendrimers (unmodified and surface partially modified with maltose and maltotriose) with anti-HIV antisense oligodeoxynucleotides. Dendriplexes obtained with unmodified G4-PPI were stable in the presence of up to 10 µg/mL concentrations of heparin and heparan sulphate and up to 1000 µg/mL of chondroitin sulphate and hyaluronic acid [195]. Dendriplexes obtained by modified G4-PPI were destroyed by physiological concentrations of heparin. An G1-PAMAM/siRNA dendriplex proved to be stable in the presence of heparin up to heparin:siRNA ratio of 1:5 (m/m) [196]. An G2-PAMAM dendrimer was derivatized with phenylboronic acid (a group with low immunogenicity and cytotoxicity) and complexed with DNA, which resulted in a stable dendriplex in serum [197]. Concerns regarding the stability of poliplexes refer to the fact that they may be disassembled by negatively charged serum proteins and, on the other hand, protein–dendriplex aggregates are rapidly eliminated from blood, leading to a low-efficacy transfection [36,198]. The stability of a PPI–siRNA dendriplex in the blood circulation was improved by the protection of the dendrimer with dithiol molecules and coverage with PEG [199].

### 3.6. The Toxicity of Dendrimers 

Numerous studies and strategies have contributed to the development of dendrimers [200,201] providing evidence on their effectiveness in therapeutic formulations. Dendrimers are monodisperse macromolecules [200], which have a compact structure with many branches, containing functional end surface groups that can easily be modified. In fact, the nature of these surface endings is the main factor that determines the toxicity of dendrimers [118].

The toxicity of dendrimers is dependent on the nature and number [118] of their functional surface groups. Therefore, changes to functional groups lead to a decrease in toxicity [202] depending on the generation and an increased compatibility with transported drugs [203].

Toxicity is dependent on the generation of PAMAM G4 < G5 < G6, the number of functional groups and the nature of terminal fragments (anionic, neutral < cationic) [200].

Although, in the first generation, the toxicity was due to the open structure and a possible interaction with biological membranes, in the case of newer-generation dendrimers, the steric nucleus prevented these interactions due to the large number of functional groups on the surface [204,205].

The toxicity profile test is the most important parameter to determine before a product is placed on the market [204]. Numerous studies, in preclinical and clinical phases, have been performed to detect toxicity and compatibility [204,206]. The modification of functional terminal groups [200] is considered of interest, particularly by reducing the cationic charge, which is directly responsible for toxicity but also leads to a reduction in therapeutic activity [200,204]. 

Amino and hydroxyl groups [205] are most often incriminated in increasing dendrimer toxicity due to penetration of the cells as a result of interaction with negatively charged lipid membranes [207]; however, in many cases, these amino groups are needed to achieve a therapeutic effect. However, modifying some terminal functional groups [208], seems to be a viable solution to these problems [200,202]. In addition to these modification techniques, the researchers explored other approaches such as the acetylation of dendrimers that leads to cationic neutralization of the amino group on the functional surface [204].

Intensive research was conducted on dendrimers to reduce their toxicity and to constantly improve their properties [200]. This showed that the dose of dendrimers also has a high impact on toxicity [203].

Moreover, researchers proposed various solutions to combat toxicity, presented in numerous publications [208]. The first approach of this kind refers to the development of dendrimers with a degradable nucleus and branching units. The proposed solutions refer to obtaining polyether dendrimers by the convergent approach (e.g., polyether imine dendrimers, polyether–copolyester dendrimers) to increase the permeability of the blood–brain barrier, phosphate dendrimers, etc. One of the best strategies refers to modifying the surface, which leads to the protection of amino groups, thus reducing cytotoxicity [209].

In recent years, there has been a considerable increase in interest in dendrimer-based drug-delivery systems [204,210], due to their high potential and biomedical applicability [201,204]. Many studies have already shown that these substances are promising for the pharmaceutical and biomedical field and intrinsic cytotoxicity can be optimized and overcome by precise chemical changes [118]. Thus, the clinical studies on dendrimers used as vectors are few [70]. For example, the efficacy and safety of a dendrimer drug delivery system based on poly-L-lysine used as nanovector for [186Re] rhenium complex coupled to an imidazolic ligand (ImDendrim) was evaluated in a clinical trial. Ten inoperable liver cancer patients that were non-responsive to conventional therapy were enrolled. The preliminary results of this study showed the safety and the effectiveness of the compound [211]. Other complexes of the dendrimer drug delivery system (DEP) and anticancer molecules are being evaluated in clinical studies: docetaxel, cabazitaxel and irinotecan [212,213]. In an open-label study, the preliminary results show a decrease in bone marrow toxicity and an enhanced efficacy in a human breast cancer model of DEP-cabazitaxel compound compared to cabazitaxel [214]. Other preclinical studies are performed to investigate the efficiency of DEP-irinotecan in human colon and pancreatic cancers. In this case, the preliminary results show a superior efficacy and survival benefits compared to standard therapies [215].

### 3.7. Toxicity Modulation Strategies

**Figure 9:**
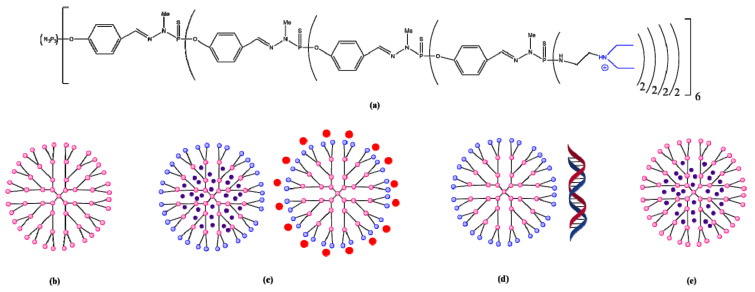
Dendrimer 6-G4, used as a support for the delivery and release of therapeutic genes (**a**) 6-G4 dendrimer; (**b**) active dendrimer; (**c**) dendrimer as drug carrier; (**d**) dendriplex; (**e**) active dendrimer as drug carrier. Adapted from [33], published by Molecules 2020.

In order to reduce the cytotoxicity of dendrimers, different structural modulation strategies have been adopted, such as:

1) PAMAM dendrimers are the most studied dendrimers, representing the dendrimers with the most important potential in gene therapy [216]. Conjugates of a siRNA antibody used in antineoplastic therapy with cationic dendrimers, PAMAM, carbosilane and phosphorus, were studied by quantitative measurements of their fluorescence intensities, zeta potential, light scattering, and dynamic scattering. The complexes were stable and had the potential to protect against nucleolytic separation of siRNA [180,217]. In antineoplastic therapy, the efficacy of transfection in HeLa and HL-60 cells was evaluated by the dendrimer–siRNA complex and the use of siRNA “cocktails”. Phosphoric dendrimers containing siRNA “cocktails” were characterized by the highest transfection rates and cytotoxicity [198,218]. Figure 9 schematically shows how to obtain conjugated phosphorus dendrimers and dendriplexes.
2) Obtaining dodecylated dendrimers: strategy used in PAMAM dendrimers of generations G2, G3 and G4 for the distribution of siRNA of luciferase-directed BCL-2 genes [219,220]. Remarkable results were obtained by dodecylated G4 dendrimers, with the best delivery efficiency, the 3D spherical shape and the introduction of gold nanoparticles in the central structure of the dendrimers being the most efficient (cell survival rate of over 90%). The ability of dendrimers to compact pDNA was greatly increased, leading to appropriately high delivery of the therapeutic gene [221]. Cyclododecylated dendrimers have been shown to be much more efficient and biocompatible than dodecylated analogs [219,222]. Moreover, the use of several radicals was studied in order to increase biocompatibility (Table 3) [222].

**Figure 10:**
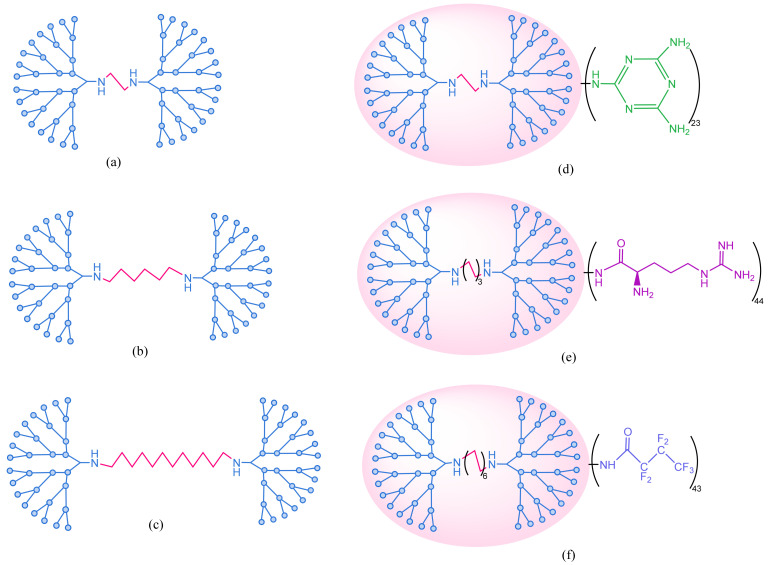
PAMAM dendrimers with a central structure consisting of diaminoethane (**a**), diaminohexane (**b**) and diaminododecane (**c**) radicals; PAMAM dendrimers obtained by modulating the surface structures of diaminoethane derivatives with triazine (**d**), diaminohexane derivatives with arginine (**e**) and diaminododecane derivatives with fluoroalkyls (**f**). Adapted from [223], published by Acta Biomaterialia, 2016.

Studies regarding gene delivery were performed to compare the effects of PAMAM dendrimers conjugated with diaminoethane, diaminohexane and diaminododecane radicals (Figure 10) [223].

The diaminododecane-cored dendrimer has the lowest nucleolar activation in transfected cells, which is a major benefit for efficient gene delivery, and the highest transfection efficiency on HEK293 cell lines (120 times higher for diaminoethane-cored dendrimers and 81 times higher for diaminohexane-cored dendrimers) [223].

The observation that the diaminododecane-cored dendrimer has minimal toxicity to transfected cells is of major importance, and is because the diaminododecane-cored dendrimer has only one lipid chain embedded inside the dendrimer, which reduces damage to the lipid fragment in cell membranes. In addition to these advantages, the diaminododecane-modified dendrimer may subsequently be subjected to different modulations. The arginine (Arg), triazine- or fluoroalkyl-modified dendrimer has superior transfection efficiency [223].

**Figure 11:**
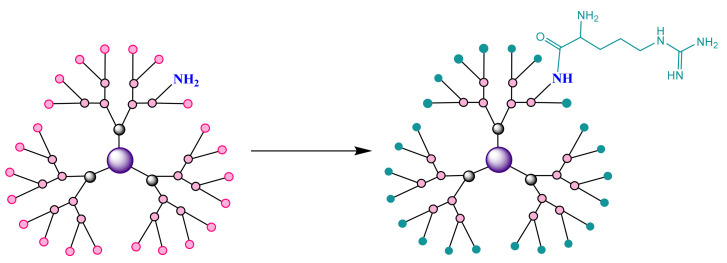
Conjugated PAMAM dendrimer with arginine to enhance siRNA delivery. Adapted from [225], published by Bioconjug Chem, 2014.

**Figure 12:**
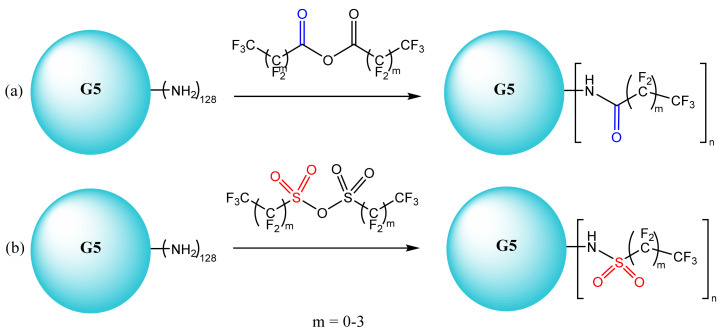
Obtaining fluorinated PAMAM dendrimers (**a**)-reaction of G5-PAMAM dendrimers with perfluoro anhydrides of carboxylic acids, (**b**)-reaction of G5-PAMAM dendrimers with perfluoro anhydrides of sulphonic acids. Adapted from [227], published by Nat Commun, 2014.

3) PAMAM G4 dendrimers with Arg terminal groups were developed as efficient nanovectors for the functional delivery of mRNA to capitalize on the unique properties of poly(amidoamine) dendrimers with triethanolamine nucleus (TEA) [223,224]. These dendrimers are structurally flexible and have been shown to be effective in delivering siRNA with numerous advantages in the cell penetration process: the 4th generation dendrimer in this category (G4Arg) has formed stable dendriplexes with siRNA, leading to an improved cellular uptake compared to its dendrimer analogue, which does not carry arginine [224]. In addition, the G4Arg dendrimer has no distinct toxicity. Thus, the addition of Arg radicals to the surface of a dendrimer has been shown to be a favorable option, with the G4Arg dendrimer (Figure 11) being an extremely promising nanovector for the efficient delivery of siRNA with high potential for other therapeutic applications [225].

4) Fluorinated dendrimers have been studied with the aim of improving transfection and reducing toxicity in gene delivery and improving the release strategy at the target site. Fluorinated PAMAM G5 dendrimers with extremely low N/P ratios (Figure 12) were tested on human embryonic kidney cells (HEK293) and human cervix-carcinoma cell (HeLa) cells. These dendrimers are characterized by serum resistance and elevated effectiveness in the process of gene transfection. G5 dendrimers are the most efficient in providing genes with a minimum toxicity, and the effectiveness of transfection depends on the degree of fluorination [226,227].

The use of fluorinated compounds in the strategy of delivery and release of therapeutic genes is based on well-studied observations. Fluoride is not present in biological systems, but is widely used due to its numerous advantages. Thus, it improves the pharmacokinetic profile and increases the therapeutic efficacy of many drugs [228,229,230], and also enhances the stability of proteins without changing their structure and functions [229]. Fluorinated compounds are both hydrophobic and lipophobic, so fluorination can help increase the affinity of polymers towards the cell membrane [231], facilitating penetration through both the lipid bilayer of the cell membrane and the endosomal/lysosomal membrane, thus facilitating endosomal penetration [232].

Fluorinated dendrimers have a low surface energy and are frequently associated with each other at low concentrations, allowing for the formation of polyplexes with NA, at low ratios of nitrogen to phosphorus (N/P) [231]. Genetic transfection at a low N/P ratio is essential to minimize toxicity to transfected cells [227].

5) In addition to PAMAM dendrimers, different generations of PPI dendrimers have been studied as genetic vectors. Thus, fluorinated PPI dendritic polymers, G3, G4 and G5, were synthesized to improve the transfection efficiency and reduce the cytotoxicity of PPI dendrimers. Fluorinated PPI dendrimers showed a higher transfection efficiency than branched poly(ethyleneimine) and Arg-modified dendrimers in HEK293 and HeLa cells [182].
6) Other structural modulations applied to dendrimers:
  (i) Dendrimers modified with folic acid, a strategy developed based on the overexpression of folic acid receptors on a variety of cancer cells, which allows for the efficient delivery of DNA and siRNA in cells overexpressing folic acid receptor [233,234].
  (ii) Dendrimers modified with nanoparticles: carbon nanotubes, graphite, gold nanoparticles, nanoparticles with different metals and silicon nanomaterials. These nanomaterials are useful due to their photothermal, magnetic and fluorescent properties [233,234,235,236].
  (iii) Dendrimers modified with magnetic nanoparticles of iron oxides (Fe_3_O_4_) or magnetic bacterial nanoparticles used for siRNA release [237,238].
  (iv) Generation 4 phosphorhydrazone dendrimer with terminal functions modified with piperidine residues (9-G4), used as a dual nVV for both a singular substance, 5-FU, and a cocktail of anti-cancer siRNA, capable of influencing downregulated anti-apoptotic genes (BCL-xL, BCL-2, MCL-1) [165,189,239]. The effect on human cervical carcinoma HeLa cells showed a considerable increase in the cytotoxic effect at low doses of the cytotoxic therapeutic agent 5-FU by complexation with cocktail dendriplexes 9-G4 / siRNA [198,240].
  (v) Introducing a fluorescent item enables the visualization of the mechanism of action. The Oregon Green modification places special emphasis on flexibility, hydrophobicity and functionality, and contributes to the high efficiency of genetic delivery [198,223,241].

## 4. Conclusions

As a promising strategy to improve specificity and reduce systemic toxicity, GDEPT therapy has been intensively studied in recent years. This strategy involves the delivery of a gene encoding an enzyme to the target site, following the selective activation of a prodrug into a cytotoxic agent. The vectors through which the gene reaches the target site contribute to the effectiveness of this strategy. Recently, there has been an increased interest in dendrimer-based delivery systems, owing to their diverse biomedical applications. Along with their many advantages, there are some limitations related to the use of dendrimers in GDEPT therapy, which requires further developments to improve the targeting and release of transgenes. The aim of this review was to provide an overview of the non-viral carriers used in the GDEPT approach, with a main focus on dendrimers, to open new perspectives and encourage further research into the design and development of prodrug-delivery vectors.

## Figures and Tables

**Table 1:** Examples of polymers used in GDEPT.

Polymer	Structure	Efficiency
PEI = Polyethylenimine or polyaziridine. Polymer with an amine group repeating unit and two aliphatic groups as spacer (CH_2_-CH_2_). They contain secondary amine groups.	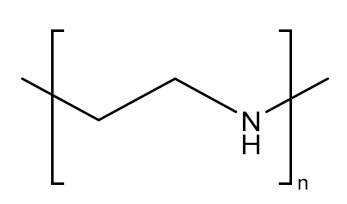	PEI is known as one of the most efficient nonviral agent currently used in GDEPT strategy [104]. The efficiency of transfection using PEI is largely due to the intense proton buffering effect and the "proton sponge effect" in the endosomal stage. At physiological pH, about 15–20% of PEI amines are protonated, which gives PEI the very intense capacity of a proton sponge [105].
Branched PEI Ethylenediamine-ethylenimine copolymer; Aziridine-1,2-diaminoethane copolymer; Ethylenediamine - ethylenimine polymer	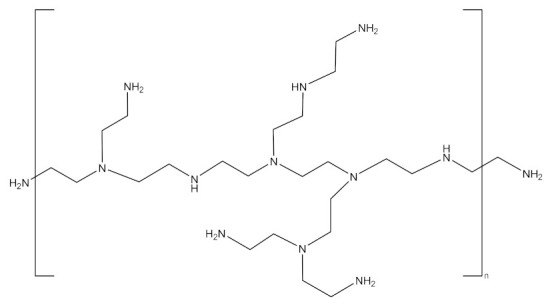	Every third atom in the PEI structure is a nitrogen atom with a very large distribution of primary, secondary and tertiary amine groups [104]. The superiority of PEI over other polymers such as PLL (lysine polypeptide) is due to the high loading density and the flexibility of the chain. PEI electrostatically condenses high molecular weight DNA in the form of polypeptic nanoparticles (10–100 nm), which are able to achieve endocytosis [106].
PPI – G4 poly(propyleneimine) G4 dendrimers with fully branched amine groups	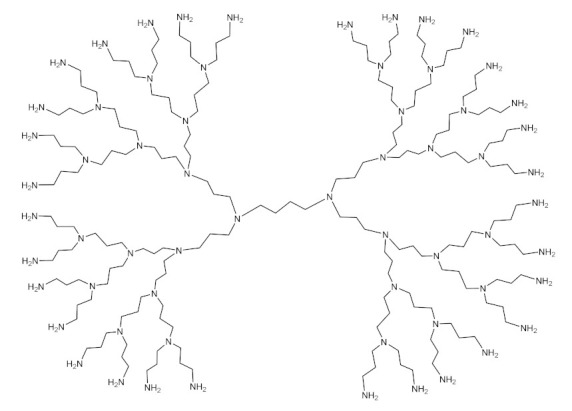	Changes in the terminal groups of the PPI dendrimer lead to a decreased toxicity, regardless of its internal structure. These structural modulations contribute to the aqueous solubility and increased stability of hydrolysis. Changes in the internal structure are also favorable by quaternization of internal tertiary amines, with the formation of cationic ammonium groups [107]. High-molecular-weight (HMW) modulations have led to a decreased cytotoxicity, while maintaining the potential for DNA binding and condensation [108].
(a) Linear poly(amidoamine) dendrimers (PAMAM); (b) Branched PAMAM dendrimers: spheroidal, cascade polymers, the size and surface charge of which determine the generation number; ethylenediamine (EDA), ammonia (NH_3_) or cystamine are used as initiating nuclei, obtaining numerous ramifications.	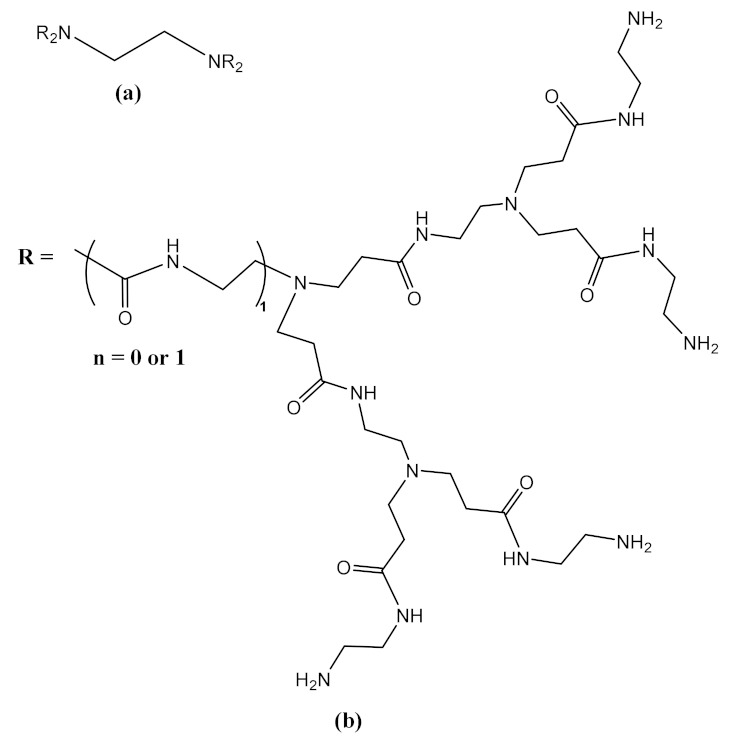	They contain a multitude of secondary and tertiary amines, which cause an intense "proton sponge" effect. PAMAM forms spherical polymers with good water solubility due to the presence of surface amine groups [109,110,111,112].
Linear PLL	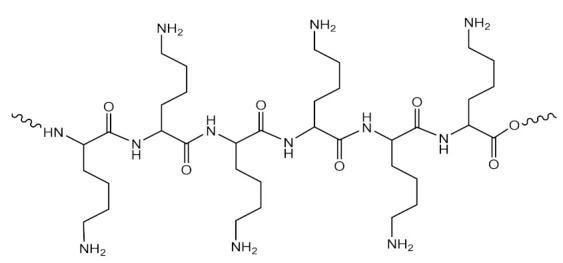	At physiological pH, amino groups are positively charged, so they have a reduced proton-buffering capacity to facilitate endosomal escape. In general, PLL has a relatively weak gene transfection activity in the absence of a lysosomal disruptive agent. In addition, unmodified in vitro PLL has increased cytotoxicity [113,114].
Branched PLL	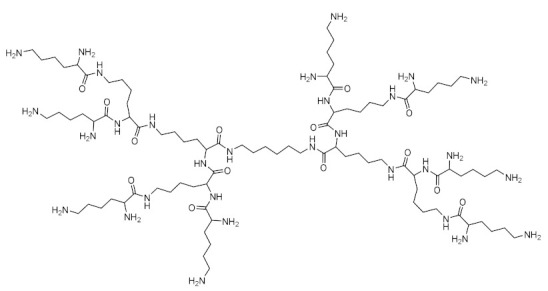	They have a large surface and branched structure, which, together with the numerous functional groups, facilitates the binding of different biological entities or molecules [115,116].
PDMAEMA poly(2-(N,N-dimethylamino)ethyl methacrylate)	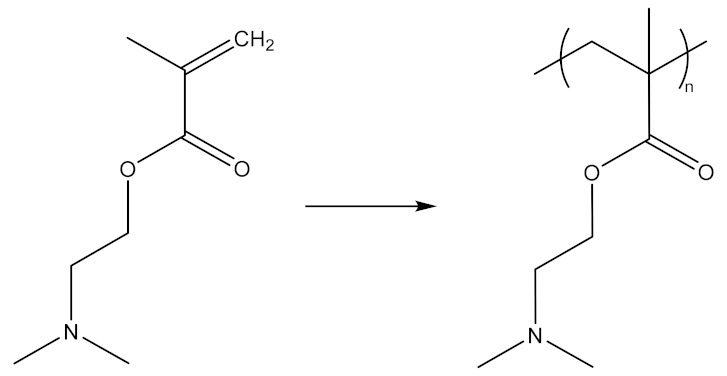	The synthesis of PDMAEMA derivatives with a molecular weight of approximately 60 kDa by controlled radical polymerization is optimal for the efficacy and toxicity of controlled transfection in GDEPT [117,118].
Chitosan	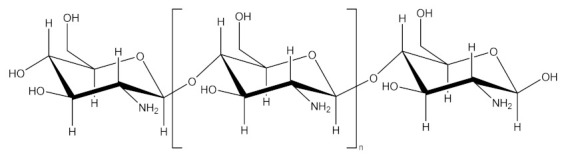	Chitosan, a natural polymer derived from chitin [119], is used as a targeting and release vector in GDEPT therapy due to its unique characteristics (cationic structure, biocompatibility, relatively low production cost and the possibility of easy functional modulation), at an optimal molecular mass between 65 and 170 kDa [120,121].
Cyclodextrin (CD)	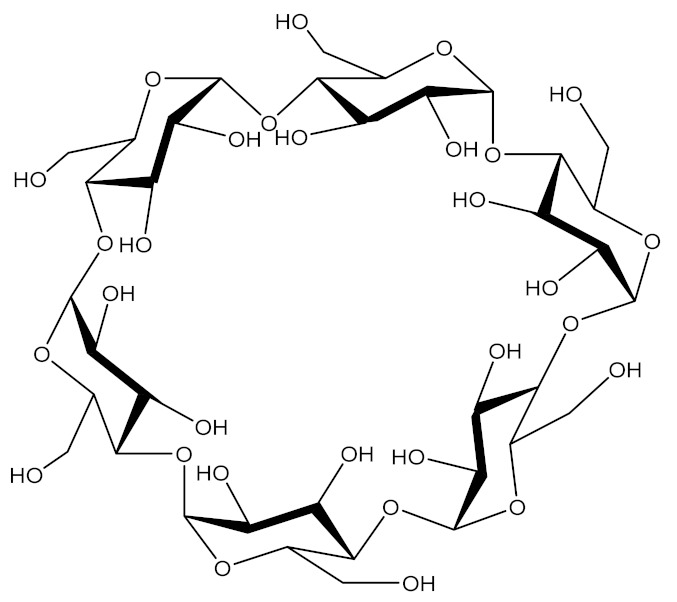	CDs generally have a limited transfection efficiency, which can be increased by various modulations/structural derivatizations: polycationic amphiphilic CDs (paCD), modification of functional groups, hydrophilic–hydrophobic balance and linker length. Another favourable modulation is represented by the modification of β-CD with a pyridylamino, alkylimidazole, methoxyethylamino or primary amine group in position 6 of the glucose units [122,123].

**Table 2:** The influence of structural modulation on dendrimers´ properties [140,149,150,151,152,153].

Advantages.	Disadvantages
**Chemical**: controllable synthesis and degradation, homogenous structure, good solubility or miscibility, numerous functional groups	**Chemical**: low solubility of higher generation dendrimers
**Pharmaceutical**: lower viscosity, multiple possibilities to bond the active substances, high bioavailability	**Pharmaceutical**: poor quality control, difficult technological transfer from research to clinical practice, tendency to aggregate in aqueous solutions
**Pharmacological**: high capacity to penetrate membranes or barriers, a good ratio between their therapeutic efficacy and toxicity, increased bioavailability and efficiency, prolonged half-life, specific activity	**Pharmacological**: superficial penetration into tissues of dendrimers with higher tendency to aggregate in aqueous solutions
	**Economic disadvantages**: higher cost of production

**Table 3:** PAMAM dendrimer pathways to increase biocompatibility and reduce cytotoxicity [222].

Radicals	Permeability	Cytotoxicity	Cellular Uptake
Acetyl	increase	decrease	
Lauroyl	increase	decrease	Increase
Amino acid	increase	increase	
PEG	decrease	decrease	Decrease

## Abbreviations

5-FU	5-fluorouracil
AAV	adeno-associated virus
ADEPT	antibody directed enzyme prodrug therapy
ARF	auxin response factors
ARF6	adenosine diphosphate-ribosylation factor 6
ASO	antisense oligonucleotides
CCL20	chemokine (C-C motif) ligand 20
CCR6	C-C motif chemokine receptor 6
CD	cyclodextrin
CEA	carcinoembryonic antigen
CRISPR	clustered regularly interspaced short palindromic repeats
CXCR4	CXC chemokine receptor-4
D	diffusion coefficient
DEP	dendrimer drug delivery system
EDA	ethylenediamine
ELAVL1	embryonic lethal abnormal vision-like 1
FA-3DNA	folic acid derivatized DNA dendrimer nanocarrier
G	generation of dendrimer
GDEPT	gene-directed enzyme prodrug therapy
GPAT	genetic prodrug activation therapy
GRAF1	GTPase regulator associated with focal adhesion kinase-1
GRP78	glucose-regulated protein
HMW	high molecular weight
HSV	herpes simplex virus
hTERT	human telomerase reverse transcriptase
HuR	human antigen R
METase	methioninase
miRNA	microRNA
mRNA	messenger RNA
N	ionizable amino groups
N/P	ratio between ionizable amino groups and phosphate groups
NA	nucleic acid
NP	nanoparticle
nVGC	non-viral vector/gene complex
nVV	non-viral vector
OC	osteocalcin
OPN	osteopontin
P	phosphate group
paCD	polycationic amphiphilic CD
PAMAM	poly(amidoamine) dendrimer
Pc	partition coefficient,
PDEPT	polymer directed enzyme prodrug therapy
PDMAEMA	poly(2-(N,N-dimethylamino)ethyl methacrylate) dendrimer
pDNA	plasmid DNA
PEG	polyethlene glycol
PEI	polyethylenimine or polyaziridine
PLL	lysine polypeptide polymer
PPI	poly(propyleneimine) polymer
PPI-G4	poly(propyleneimine) G4 dendrimers
RG7388	selective MDM2 inhibitor that blocks the binding of MDM2
SCID	severe combined immunodeficiency
siRNA	short-interfering RNA
TEA	triethanolamine
TNFα	tumor necrosis factor alpha
VDEPT	virus directed enzyme prodrug therapy
VV	viral vector
